# Decreased biofilm formation in *Proteus mirabilis* after short-term exposure to a simulated microgravity environment

**DOI:** 10.1007/s42770-021-00588-y

**Published:** 2021-09-23

**Authors:** Dapeng Wang, Po Bai, Bin Zhang, Xiaolei Su, Xuege Jiang, Tingzheng Fang, Junfeng Wang, Changting Liu

**Affiliations:** 1 Department of Respiratory and Critical Care Medicine, The Second Medical Center & National Clinical Research Center for Geriatric Disease, Medical School of Chinese PLA, Beijing, People’s Republic of China; 2 Respiratory Diseases Department, PLA Rocket Force Characteristic Medical Center, Beijing, People’s Republic of China; 3 Respiratory Diseases Department and Critical Care Medicine, Binzhou Medical University Hospital, Binzhou, People’s Republic of China; 4 Respiratory Diseases Department, The Second Medical Center of PLA General Hospital, Beijing, 100853 People’s Republic of China

**Keywords:** Simulated microgravity, *Proteus mirabilis*, High-aspect rotating vessel, Biofilm formation, Transcriptome sequencing

## Abstract

**Background:**

Microbes threaten human health in space exploration. Studies have shown that *Proteus mirabilis* has been found in human space habitats. In addition, the biological characteristics of *P. mirabilis* in space have been studied unconditionally. The simulated microgravity environment provides a platform for understanding the changes in the biological characteristics of *P. mirabilis*.

**Objective:**

This study intends to explore the effect of simulated microgravity on *P. mirabilis*, the formation of *P. mirabilis* biofilm, and its related mechanism.

**Methods:**

The strange deformable rods were cultured continuously for 14 days under microgravity simulated in high-aspect rotating vessels (HARVs). The morphology, growth rate, metabolism, and biofilm formation of the strain were measured, and the phenotypic changes of *P. mirabilis* were evaluated. Transcriptome sequencing was used to detect differentially expressed genes under simulated microgravity and compared with phenotype.

**Results:**

The growth rate, metabolic ability, and biofilm forming ability of *P. mirabilis* were lower than those of normal gravity culture under the condition of simulated microgravity. Further analysis showed that the decrease of growth rate, metabolic ability, and biofilm forming ability may be caused by the downregulation of related genes (*pstS*, *sodB*, and *fumC*).

**Conclusion:**

The simulated microgravity condition enables us to explore the potential relationship between bacterial phenotype and molecular biology, thus opening up a suitable and constructive method for medical fields that have not been explored before. It provides a certain strategy for the treatment of *P. mirabilis* infectious diseases in space environment by exploring the microgravity of *P. mirabilis*.

**Supplementary Information:**

The online version contains supplementary material available at 10.1007/s42770-021-00588-y.

## Introduction

Manned space technology is developing rapidly. Astronauts and cabin equipment inevitably bring microbes, including bacteria, fungi, and viruses, into space in the process of manned spaceflight. The space environment is characterized by a series of prominent features more than 100 km above the earth’s surface, including strong ionizing radiation, high vacuum, ultra-low temperature, ultra-high temperature, and weightlessness [1–3]. Various factors in the space environment can induce genetic changes of microorganisms, and then affect the phenotypic characteristics of microorganisms, including morphology, growth rate, biofilm forming ability, virulence, and drug resistance, which may induce infectious diseases and affect the health of resident personnel. As a consequence, it is of great significance to study the phenotypic characteristics of microorganisms in space environment.

*Proteus mirabilis* belongs to *Proteus* and widely exists in soil, water, and feces [4]. In addition, *P. mirabilis* is highly pathogenic and is one of the main causes of persistent and refractory urinary tract infections. Studies have shown that *P. mirabilis* has been tested for in space and post-flight astronaut samples in human habitats [5, 6]. The occurrence of infectious diseases is more likely to occur due to the decline of human immunity in the space environment. As a consequence, how to prevent and treat the infection caused by *P. mirabilis* in the space environment is worthy of attention. However, studies on the biological characteristics of *P. mirabilis* in the space environment have not been carried out due to concerns about the cost, time, and safety of spacecraft flight.

Microgravity environment refers to the environment in which the apparent weight of the system is much smaller than its actual weight under the action of gravity. In fact, it is the most important environmental factor encountered in the process of manned spaceflight. Microgravity simulation experiment has become an important and necessary preparation for the development of manned spaceflight. The biological characteristics of microorganisms under simulated microgravity have been widely studied in recent years. Some results show that under the simulated microgravity conditions, microorganisms show the same biological characteristics as the space environment. For example, it was found that the virulence of *Salmonella typhimurium* increased under simulated microgravity, which was consistent with that of *Salmonella typhimurium* under space microgravity. Further studies at the molecular level also show that RNA-binding protein Hfq is involved in the process of simulating the effects of microgravity and space environment. A simulated microgravimeter was used to observe the changes in biological characteristics and internal mechanism of *P. mirabilis* under microgravity. It can provide a strategy for the prevention and treatment of infectious diseases caused by *P. mirabilis* in space environment in the future.

## Materials and methods

### Bacterial strains and culture conditions

The whole study used *P. mirabilis* clinically separated from the urine of a male patient. The API20NE identification system (BioMèrieux, Craponne, France) and 16S rDNA gene sequencing were applied to identify the strains. The *P. mirabilis* strains grew aerobically in Luria-Bertani (LB) medium (with 0.5% agar) at 37 °C. The experimental (simulated microgravity, SMG) and the normal gravity (NG) control were set up in a high-aspect rotating vessel (HARV) bioreactor (Synthecon, Inc., Houston, TX, USA) and cultured continuously. Supplementary Figure 1 shows SMG culturing by rotating the bioreactor with the axis perpendicular to gravity, while the cultivation under NG is realized in case of its axis parallel to gravity [7]. *P. mirabilis* was inoculated in the HARV bioreactor with a dilution of 1:200. Each bioreactor was fully filled with fresh LB medium of ~55 ml, and the bubbles were removed carefully. After culturing in HARVs at 37 °C for 24 h at 25 rpm, the *P. mirabilis* cultures under SMG and NG were diluted and transferred into another HARVs fully filled with LB medium. *P. mirabilis* inoculation was cultured in the HARV bioreactors for 2 weeks. *P. mirabilis* cultured under SMG was called PMML strain, while that cultured under NG was called PMGL strain. The PMML and PMGL groups were serially diluted in phosphate-buffered saline (PBS), plated on LB agar, and counted. The cultures obtained were tested in various ways.

### Phenotypic analysis

#### Scanning electron microscopy (SEM)

PMML and PMGL strains were cultured in LB medium, washed with sterile phosphate-buffered saline (PBS; pH 7.4), and fixed with 4% glutaraldehyde overnight. The samples were washed again with PBS, dehydrated in increasing grades of ethanol, and then critical pointdried. After coating the specimens with gold-palladium, they were observed using an FEI Quanta 200 scanning electron microscope (USA).

#### Growth rate assay

The Bioscreen C system and BioLink software (Lab Systems) were applied to monitor *P. mirabilis* growth. The overnight growing of *P. mirabilis* strains was made in LB medium at 37 °C. Twenty microliters of the overnight culture at a concentration of 10^6^ CFU/ml was cultivated in Bioscreen C 96-well microtiter plates (Lab Systems). The plates containing fresh LB growth medium of 350 μl per well were incubated under continuous shaking at the maximum amplitude for 24 h at 37 °C. The OD_630_ was measured every 2 h. *P. mirabilis* growth experiments were performed in triplicate.

#### Carbon source utilization and chemical sensitivity assay

The biochemical features of two strains were tested by using the Biolog GENIII MicroPlate (Biolog, CA) and 94 phenotypic tests, including utilization of 71 carbon sources and assays of 23 chemical sensitivity. The overnight growing of *P. mirabilis* strains on agar plates was made at 37 °C. The centrifugal tube containing two strains was washed with PBS and inoculated into the IF-A inoculum (Biolog, CA). A turbidimeter was used to adjust the concentration of *P. mirabilis* suspension to 10^8^ CFU/ml, and the suspension of 100 μl was inoculated into each well of the 96-well plate. After 24-h incubation at 37 °C, an automated Biolog microplate reader at 630 nm was used to measure the absorbance. The experiment was carried out in triplicate.

#### Biofilm assay

##### Crystal violet staining (a)

After being diluted (1:100) in 5-ml LB medium, the PMML and PMGL cultures were moved to glass tubes and incubated at 37 °C for 24 h at 200 rpm. The planktonic bacteria were removed. Soon afterwards, deionized water was used to wash each tube three or four times. Then, 0.1% of crystal violet dye was used to stain the glass tubes for 15 min at 37 °C. The experiment was carried out in triplicate.

##### Crystal violet staining (b)

After adding approximately 200 μl of the *P. mirabilis* suspension culture (1–5 × 10^7^ CFU/ml) to 96-well microculture plates, the mixture was incubated at 37 °C for 24 h. PBS was used to wash the wells two times. In total, 200 μl of 0.1% crystal violet was employed to stain the cells (Sigma, St. Louis, MO, USA) for 30 min and washed with PBS. Next, the microculture plates were dried, and the dissolution of the stained biomass samples in 95% ethanol was made. The Thermo Multiskan Ascent Instrument (Thermo, USA) was applied to determine OD_570_ for each well. The experiments were carried out in triplicate.

##### Analysis of biofilm formation ability via confocal laser scanning microscopy (CLSM)

The two strains were cultured on LB medium overnight and inoculated in 35-mm confocal dishes (Solarbio, Beijing, China). The plates were incubated at 37 °C for 24 h and stained with a Filmtracer LIVE/DEAD Biofilm Viability Kit (Invitrogen, Carlsbad, CA, USA) in accordance with the manufacturer’s instructions. Three image stacks were obtained from each sample randomly by CLSM using a Leica TCS SP8 microscope with a Lecia TCS SP8 CSU imaging system. All the experiments were carried out in triplicate.

#### Transcriptomic sequencing and comparison

##### Sequencing and filtering

*P. mirabilis* cells were collected after 5-min centrifugation at 8000 × *g* at 4 °C. The RNeasy Protect Bacteria Mini Kit (Qiagen, Germany) was used to extract total RNA from the two strains according to the manufacturer’s instructions. The 10-min centrifugation of *P*. *mirabilis* was made at 10,000 × *g* at 4 °C. Chloroform added with the supernatant was mixed for 15 s. After being transferred into a tube filled with isopropanol, the upper aqueous phase was centrifuged at 13,600 × *g* for 20 min at 4 °C. After removing the supernatant, 3-min centrifugation of the mixture of precipitate and ethanol was made at 12,000 × *g* and 4 °C. Soon afterwards, the supernatant was discarded, and the 20-s centrifugation of the sample was made at 12,000 × *g* and 4 °C. After removing the residual liquid by air-drying, the dissolution of RNA pellet in RNase-free water was made. The purity of the samples is tested by NanoDrop™. Divalent cations were used to fragment the purified mRNAs into small pieces (~200 bp). The first strand of cDNA using reverse transcriptase and random primers was generated by applying RNA fragments. Following this, DNA polymerase I and RNase H were used to create the second strand of cDNA. The enrichment and quantification of cDNA fragments were made by using PCR amplification and Qubit 2.0, respectively. Lastly, the BGISEQ-500 was used to construct and sequence cDNA libraries. The SOAPnuke software (version 1.5.2) was applied to filter raw reads, and adapter reads and poly N reads were removed from the raw data to acquire clean reads. Clean reads were located to the reference genome by Bowtie2-2.2.3 [8]. Gene expression was quantified by HTSeq v0.6.1, and the calculation of the fragments per kilobase of transcript per million mapped reads (FPKM) of each gene was made according to the gene length. Lastly, the mapping of read counts to the genes was made. The DESeq R package (1.18.0) was employed to make differential gene expression analysis of the two strains. Through DESeq analysis, it is considered that genes yielding a *p* value of < 0.05 are differentially expressed. The KEGGseq R package was applied to analyze Kyoto Encyclopedia of Genes and Genomes (KEGG) enrichment of differentially expressed genes (DEGs). It was considered that DEGs with a *p* value of < 0.05 are statistically significant. KOBAS software was used to assess the statistical enrichment of DEGs in KEGG pathways.

##### Statistical analysis of gene expression values and DEGs

After mapping the clean reads to the reference strain by applying HISAT (version 2.0.4) and Bowtie2 (version 2.2.5) [9], the gene expression level with RSEM [8] was calculated. Cluster software (version 3.0) was applied to make cluster analysis of gene expression, and Java TreeView (3.0) [10] was used to visualize the results of cluster analysis.

##### Functional annotation and enrichment analysis

The genes from the reference sequences in the KEGG database were selected using the BLAST software to compare and annotate gene function. The gene expression profiles of different sample groups were analyzed using cluster analysis. The transcriptome data was analyzed by identifying DEGs according to the KEGG pathway. Gene enrichment analysis was performed using KEGG functional annotation. A *p* value of ≤ 0.05 indicated significant enrichment of DEGs.

#### Quantitative real-time PCR (qPCR)

Total RNA and random hexamer primers with SuperscriptIII reverse transcriptase (Invitrogen) using qPCR were employed to synthesize cDNA. The StepOnePLUS PCR system (Thermo, USA) with cDNA as the template was used to carry out the experiment in duplicate for each RNA sample. The related fold change of the target genes in the test and controlled RNA samples was determined. The 16S rRNA gene was regarded as an internal reference. The primer sequences used in this study are shown in Table 1.

**Table 1:** Primers for the target genes used in qRT-PCR

Gene name	Size (bp)		Primer sequences (5q to 3′)
16S	104	Forward	TCACCTAGGCGACGATCTCT
		Reverse	GTGCAATATTCCCCACTGCT
PMI_RS06255	111	Forward	GAACCAAACCGTGAGCGTAT
		Reverse	AGCTTTCTTCGCGATCTCTG
PMI_RS06750	94	Forward	TGGTTCAGGTTGGACTTGGT
		Reverse	ATCTTCGCCAGAAACAGGTG
PMI_RS12550	105	Forward	CAGGTTAGCTCCGGAAAGTG

### Statistical analysis

The quantitative experiments were performed in triplicate and the data were represented as the mean ± standard deviation (S.D.). A statistical comparison of the data was conducted using two-tailed Student’s *t*-test. Statistical significance between the two groups was defined as *p* ≤ 0.05.

## Results

### Phenotypic characteristics

#### Electron microscopy findings

**Fig. 1:**
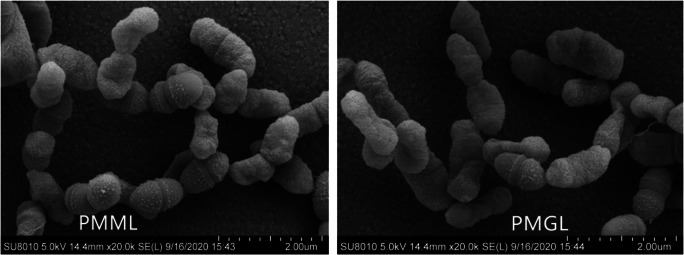
Experimental setup of the high-aspect rotating vessel bioreactors. *P. mirabilis* cells in the HARV bioreactor are grown under the simulated microgravity (SMG) condition with its axis of rotation perpendicular to gravity or grown under the normal gravity (NG) with its axis of rotation vertical to gravity. The bioreactors are filled with LB medium and the bubbles are removed

Scanning electron microscopy was performed to observe single-cell morphology in the PMML and PMGL strains. The results showed that the PMML had no changes in intercellular mucus and had smooth cell walls than the PMGL strains (Fig. 1).

#### Growth rate assay

**Fig. 2:**
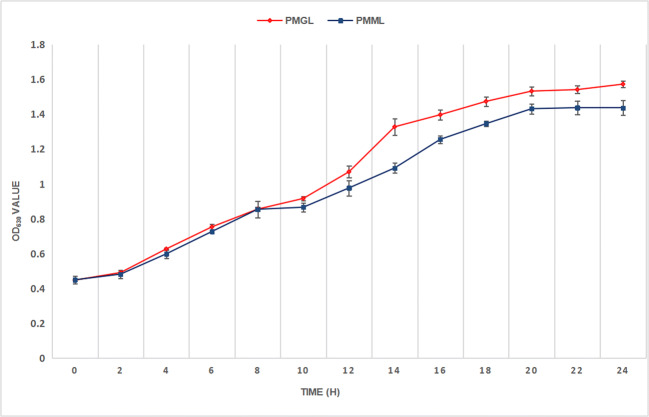
Scanning electron micrographs (SEM) of PMML and PMGL strains. **a** SEM of the PMML strain. **b** SEM of the GS1 strain

A slight difference was observed between the two strains in terms of growth rate with time (Fig. 2, Table 2). When compared with PMGL, the PMML strain exhibited a decreased growth rate, especially after 14 h (*p* = 0.0095).

**Table 2:** OD_630_ values of PMML and PMGL

Time (h)	PMML	PMGL	*p* value
0	0.4497±0.0222	0.4480±0.0066	0.873
2	0.4823±0.0232	0.4913±0.0047	0.627
4	0.5987±0.0264	0.6270±0.0062	0.251
6	0.7270±0.0066	0.7537±0.0168	0.172
8	0.7780±0.0476	0.8553±0.0110	0.183
10	0.8663±0.0262	0.9167±0.0131	0.155
12	0.9770±0.0439	1.0697±0.0343	0.018
14	1.0917±0.0280	1.3280±0.0485	0.031
16	1.2560+0.0216	1.3973±0.0279	0.010
18	1.346±0.0147	1.4740±0.0269	0.026
20	1.4320±0.0293	1.5330±0.0246	0.021
22	1.4380±0.0397	1.5417±0.0216	0.024
24	1.4370±0.0416	1.5730±0.0180	0.023

#### Chemical sensitivity and carbon source utilization assays

**Fig. 3:**
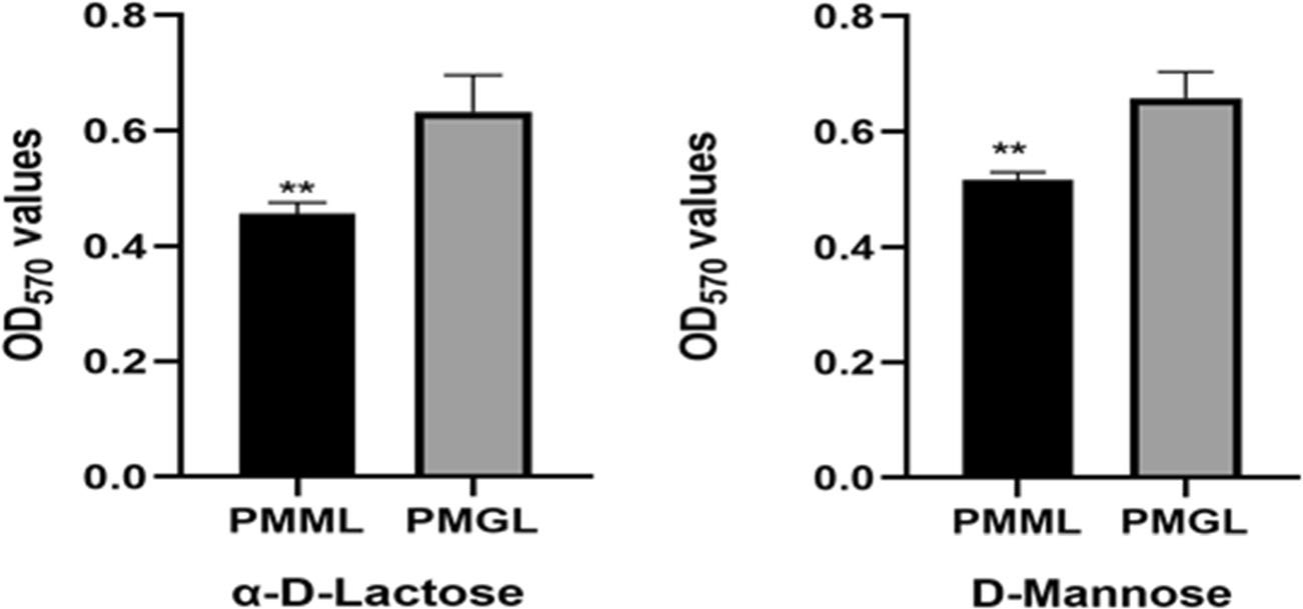
Growth curves of two *P. mirabilis* strains. Growth curves of PMML (blue) and PMGL (red) were determined by measuring the OD_630_ value, which represents the bacterial concentration. The OD_630_ value was measured every 2 h for 24 h

The chemical sensitivity assay showed decreased utilization of α-d-lactose and d-mannose (*p* = 0.0226 and *p* = 0.046, respectively) by PMML compared to that by PMGL (Fig. 3). It is speculated that *P. mirabilis* adapts to a new environment by changing the characteristics of its metabolism, as seen in the simulated microgravity environment. However, the mechanisms involved in this metabolic change are still unclear. Carbon source utilization assays showed that there were no significant differences between other metabolites and stress response pores. However, the Biolog experiment of this study was completed on ground, and not in an actual space environment and, therefore, requires further exploration.

#### Biofilm assay

##### The biofilm forming ability of *P. mirabilis*

**Fig. 4:**
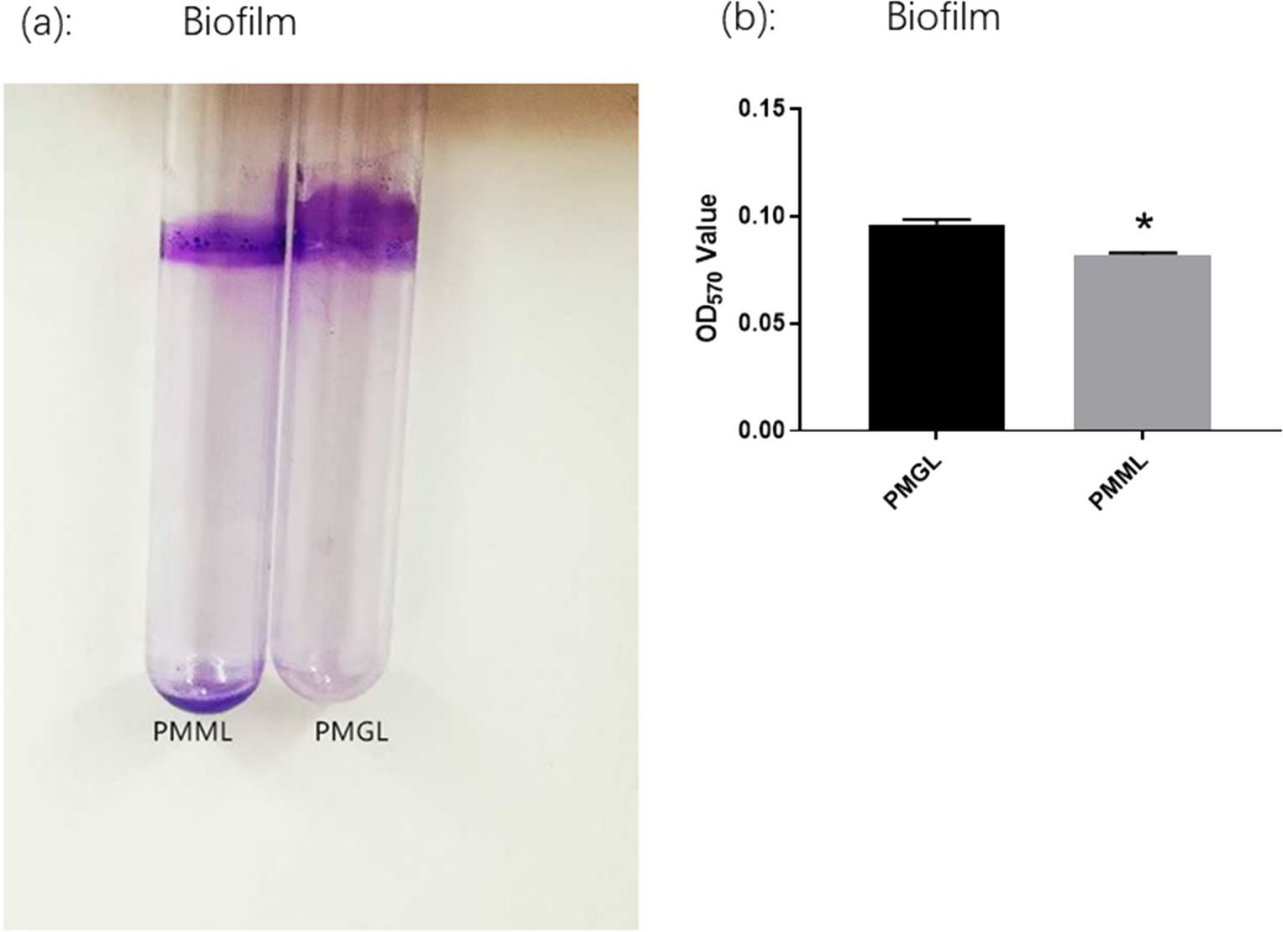
Chemical sensitivity assays of the two strains. The PMML and PMGL strains were incubated in a Biolog GENIII MicroPlate at 37 °C for 24 h in LB medium and tested for carbon source utilization and chemical sensitivity. The effect of simulated microgravity (SMG) on the expression of α-d-lactose (**a**) and d-mannose (**b**) is shown. Asterisk indicates significant difference at *p* < 0.05

The biofilm forming ability of *P. mirabilis* is considered a significant phenotype. This ability was analyzed in the PMML and PMGL cultures after a 2-week cultivation. The biofilms formed on the glass tubes were monitored by crystal violet staining (a). OD_570_ was measured after crystal violet staining (b). The adhered pellicles were further quantified using the OD_570_ values. PMGL showed a higher biofilm forming ability than PMML (*p* = 0.0022) (Fig. 4).

##### Analysis of biofilm formation ability of *P. mirabilis* strain using CLSM

**Fig. 5:**
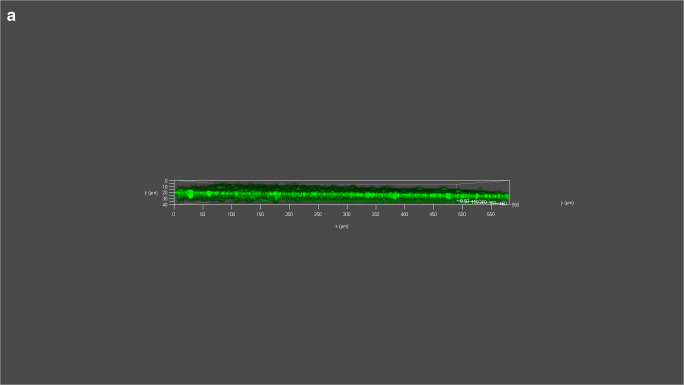

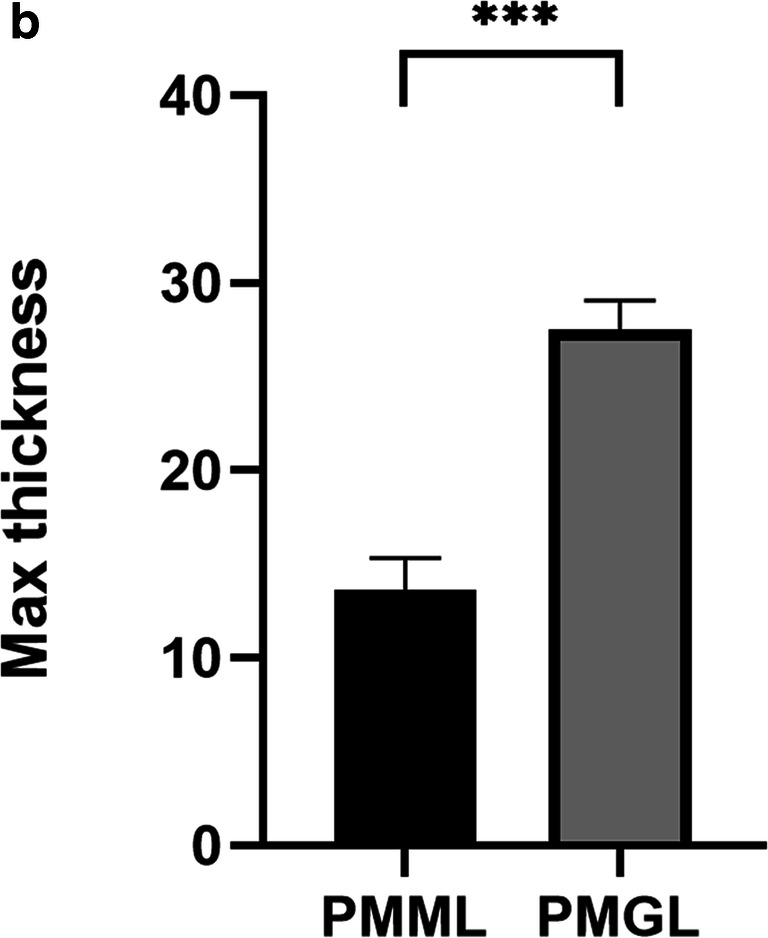
Analysis of biofilm formation ability via crystal violet staining. The PMML and PMGL strains were cultured in 96-well polystyrene microtiter plates at 37 °C for 24 h. Biofilm forming ability was measured. The biofilms formed on the glass tubes were analyzed using crystal violet staining (**a**) and quantified by measuring the absorbance of crystal violet at 570 nm (**b**)

Biofilms of PMML and PMGL strains were cultured in 35-mm confocal dishes. Cells were stained with a Filmtracer LIVE/DEAD Biofilm Viability Kit, and biofilm formation ability was tested using confocal laser scanning microscopy (CLSM). Green fluorescence indicates live cells; red fluorescence indicates dead cells. “***” represents adjusted *p* value <0.01; “*”represents adjusted *p* value <0.05. (a) Analysis of biofilm formation ability of PMML strain using CLSM. (b) Analysis of biofilm formation ability of PMGL strain using CLSM. (c) Analysis of biofilm formation ability of PMML and PMGL strain using CLSM (Fig. 5; Supplementary Figure 2).

### RNA-Seq mapping and comparative genomic analysis

According to the functional analysis of the KEGG pathway, all DEG clusters were analyzed. Both upregulated and downregulated genes were identified. In PMML, approximately 12 genes were upregulated, and 17 genes were downregulated (Supplementary Figure 3).

We found that the downregulated genes outnumbered the upregulated genes, suggesting that gene metabolism expression was inhibited in PMML. Additionally, most of the DEGs were associated with amino acid metabolism, sugar metabolism, transport, and signal transduction (Supplementary Figure 4; Table 3). Three DEGs related to biofilm metabolism were identified in PMML, including PMI_RS12550 (*pstS*), PMI_RS06750 (*sodB*), and PMI_RS06255 (*fumC*). The above results were verified using qPCR.

**Table 3:** Details of the differentially expressed genes (DEGs)

Gene ID	Designation	Length (bp)	log2FoldChange (PMML/PMGL)	Up/down	*p* value	Gene function
PMI_RS14830		2495	1.42386006927969	Up	6.96E-05	Mat/Ecp fimbriae outer membrane usher protein
PMI_RS04770		543	1.08047931271713	Up	0.00016	Uridine kinase
PMI_RS08220		603	1.7635152260287	Up	2.50E-05	LuxR family transcriptional regulator, capsular biosynthesis positive transcription factor
PMI_RS10390	mgtE	1074	1.61430565477922	Up	3.82E-06	Fimbrial chaperone protein
PMI_RS12550		699	1.67510731358992	Up	3.82E-06	Fimbrial chaperone protein
PMI_RS09675		861	1.84898299118261	Up	7.76E-05	No
PMI_RS14835		864	1.45666073453672	Up	4.40E-05	Mat/Ecp fimbriae periplasmic chaperone
PMI_RS09275		765	1.31823468916688	Up	0.00043	Chaperone protein PapD
PMI_RS09270		2487	1.53135272820167	Up	2.42E-05	Outer membrane usher protein PapC
PMI_RS17505			1.31232107003372	Up	0.000109	XRE family transcriptional regulator, master regulator for biofilm formation
PMI_RS15440		1353	1.70558100358398	Up	0.00032	No
PMI_RS03790	pyrD	1011	1.10139831452801	Up	8.69E-05	Type VI secretion system secreted protein Hcp
PMI_RS06685	gstA	612	−1.51757779099496	Down	0.00028	Lglutathione S-transferase
PMI_RS09365	grcA	384	−1.7488696118053	Down	6.98E-05	Autonomous glycyl radical cofactor
PMI_RS01070		1383	−1.36339040837153	Down	6.35E-05	Solute carrier family
PMI_RS18490	gpmB	648	−1.46649446000024	Down	7.08E-05	Probable phosphoglycerate mutase
PMI_RS13405		279	−1.90346273574243	Down	0.00028	Ethanolamine utilization protein EutM
PMI_RS03835	rmf	171	−1.77234261558484	Down	0.00025	Ribosome modulation factor
PMI_RS06255	fumc	1398	−1.53400675009522	Down	0.00031	Fumarate hydratase, class II
PMI_RS04910		243	−1.46880616244531	Down	0.00036	No
PMI_RS12610		1071	−1.35999327826646	Down	6.86E-06	Integrase/recombinase XerC
PMI_RS06750	sodB	579	−1.11371491034908	Down	9.33E-05	Superoxide dismutase
PMI_RS19020	ssrA	367	−2.3513452021666	Down	2.07E-06	No
PMI_RS14315	pstB	777	−1.91031855481805	Down	0.00043	Chaperone protein PapD
PMI_RS06815		240	−1.77700473582651	Down	0.00015	Murein lipoprotein
PMI_RS05420		519	−1.5049377019371	Down	1.91E-05	Secretion system secreted protein Hcp
PMI_RS05240	ychH	279	−2.48872258916291	Down	7.89E-07	No
PMI_RS14300	pstS	1041	−1.88909737452914	Down	0.00025	Phosphate transport system substrate-binding protein
PMI_RS03690		519	−1.50265019782485	Down	8.69E-05	Type VI secretion system secreted protein Hcp

### mRNA expression levels of key genes related to biofilm formation

**Fig. 6:**
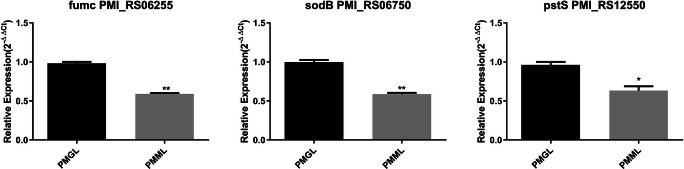
Analysis of biofilm formation ability via CLSM. **a** Analysis of biofilm formation ability of PMML strain using CLSM. **b** Analysis of biofilm formation ability of PMGL strain using CLSM. **c** Analysis of biofilm formation ability of PMML and PMGL strain using CLSM

The mRNA expression levels of key genes related to biofilm formation were analyzed to determine the mechanisms of biofilm formation by PMML and PMGL. PMML exhibited decreased *pstS*, *sodB*, and *fumC* levels compared to PMGL (*p* = 0.025, *p* = 0.0056, and *p* = 0.004, respectively) (Fig. 6).

## Discussion

*P. mirabilis* was exposed to simulated microgravity for 14 days using HARV. In addition, its phenotypic characteristics, including morphology, growth rate, metabolism, and biofilm formation ability, were analyzed. *P. mirabilis* cultured under the condition of SMG showed a decrease in growth rate, metabolic ability, and biofilm formation ability. Further analysis showed that the decrease of biofilm forming ability may be related to the downregulated expression of genes related to biofilm formation (*pstS*, *sodB*, and *fumC*). It is the first study of *P. mirabilis* in a SMG environment, providing a certain basis for the prevention and treatment of *P. mirabilis* infectious diseases in the future space environment.

Compared with PMGL, the proliferation of PMML was significantly slower than that of PMGL in the logarithmic growth phase. In addition, the growth rate of PMML decreased, which may be related to the downregulation of the genes involved and changes in metabolism and ribosomal structure. Previous studies have also observed changes in the same rate of growth [11]. In addition, Nicholson et al. [12] analyzed the growth rate of *Bacillus subtilis* spores on the O/OREOS spacecraft. The flying group of *B. subtilis* cells also showed a lower growth rate compared with the ground control experiment.

The regulation of bacterial metabolism may involve many aspects, including changes in growth rate, utilization of nutrients, and changes in metabolites in a complex environment. Studies have shown that α-d-lactose and d-mannose provide necessary energy support in the process of biofilm formation [13–16]. In this study, it was found that in the simulated microgravity environment, the utilization rate of two carbon sources (α-d-lactose and d-mannose) decreased. However, the decrease in the utilization rate of α-d-lactose and d-mannose may slow down the biofilm formation or reduce the intensity of biofilm formation. As a result, it further affected the biofilm formation and reduced the growth rate of *P. mirabilis.*

The biofilm formation in bacteria was first carried out under the action of microgravity in 2001 [17]. Three genes related to biofilm formation, including *pstS*, *sodB*, and *fumC*, were downregulated in SMG. In theory, *pstS* is a factor related to the ability of bacteria to form biofilm [18]. Some people think that the formation of bacterial biofilm may be induced by *sodB* [19]. Gabryszewski et al. confirmed the existence of *fumC* in biofilm and its ability to promote biofilm formation [20]. It has been found that *pstS*, *sodB*, and *fumC* may affect the growth rate of bacteria [21–23]. Studies have shown that the reduction of biofilm formation will affect the viability of bacteria and reduce the growth rate of bacteria [21]. A study reports that *pstS* is involved in the formation of biofilms [24]. The main genes involved in the formation of bacterial biofilm may be *pstS* [25, 26]. We believe that due to the stimulation of microgravity, the expression of *pstS* is downregulated, the biofilm forming ability is decreased, and the biofilm becomes thinner, which may affect the growth rate of *P. mirabilis*. Hassett et al. observed that *sodB* significantly affected the growth rate of *Pseudomonas aeruginosa* [22]. *SodB* is a factor related to biofilm formation, and the decrease of *sodB* expression will reduce the biofilm forming ability of bacteria [27]. In *P. mirabilis*, the environmental changes that inhibit the activity of *sodB* also lead to the weakening of biofilm, which may reduce the growth rate of *P. mirabilis*. Studies have shown that the growth rate of bacteria is affected by the expression of *fumC* gene [23]. *Fumc* is a kind of II enzyme, which is related to the formation of biofilm [28]. In our opinion, the decrease of *fumC* expression may weaken the formation of biofilm, resulting in a decrease in the growth rate of *P. mirabilis*. As a result, it is speculated that the decrease in the growth rate of *P. mirabilis* under SMG may be attributed to the downregulation of genes related to biofilm formation.

The growth of bacteria on the surface of microorganisms and the ability of biofilm formation under different growth conditions are affected by many factors, including the type of culture, cutting methods, and nutrient utilization. As a result, it showed that *Pseudomonas aeruginosa* showed increased biofilm formation and new structure formation during spaceflight [29]. In addition, studies have shown that spaceflight reduces the biofilm forming ability of the multidrug-resistant *Acinetobacter baumannii* [30]. Studies have shown that the uncertainty in the treatment of *P. mirabilis* is closely related to the intensity of biofilm formation [31]. Crystal violet staining and confocal scanning electron microscopy were used to detect the biofilm forming ability of *P. mirabilis*. Compared with PMGL, the biofilm forming ability of PMML decreased. For further analysis at the transcriptional level, a series of biofilm forming–related genes were detected in the SMG group. As a result, the decrease of biofilm formation ability of *P. mirabilis* in the SMG environment could be attributed to the downregulation of the expression of the above genes.

## Conclusion

The study provided data showing a decline in growth rate, metabolism, and biofilm formation of *P. mirabilis* after 14 days of exposure to simulated microgravity. In addition, further analysis showed that the decrease of growth rate, metabolic ability, and biofilm forming ability may be due to the downregulation of biofilm formation and synthesis (*pstS*, *sodB*, and *fumC*) gene expression. The SMG condition enables us to explore the potential relationship between bacterial phenotype and molecular biology, thus opening up a suitable and constructive method for medical fields that have not been explored before. It provides a certain strategy for the treatment of *P. mirabilis* infectious diseases in space environment by exploring the microgravity of *P. mirabilis*.

## Supplementary information

ESM 1 (JPG 109 kb)

ESM 2 (PNG 898 kb)

High Resolution Image (TIF 6079 kb)

ESM 3 (PNG 121 kb)

ESM 4 (PNG 108 kb)

ESM 5 (PNG 154 kb)

ESM 6 (PNG 143 kb)
