# Supplementary figures and images for: Decreased biofilm formation in Proteus mirabilis after short-term exposure to a simulated microgravity environment
Source: Braz J Microbiol. 2021 Sep 23;52(4):2021–30. doi: 10.1007/s42770-021-00588-y (PMC8578233; doi:10.1007/s42770-021-00588-y)

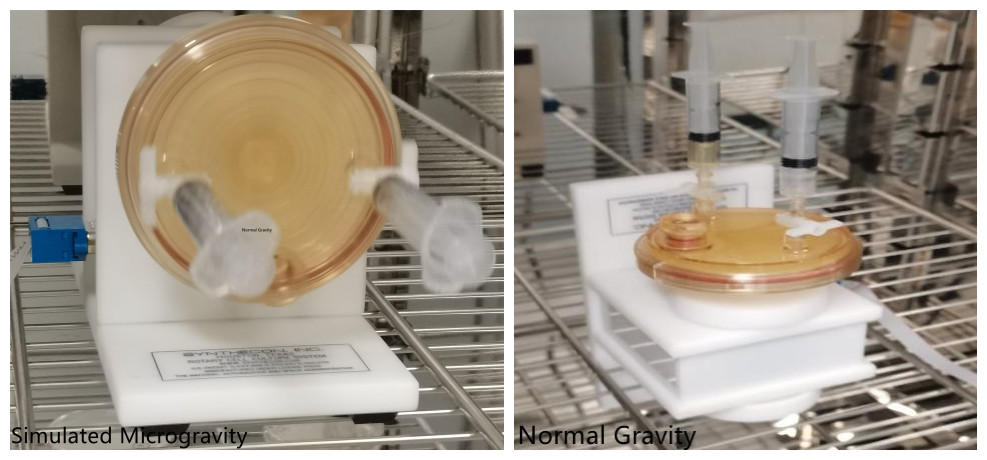

Supplement: Supplementary file 1 — (JPG 109 kb) [file 42770_2021_588_MOESM1_ESM.jpg]

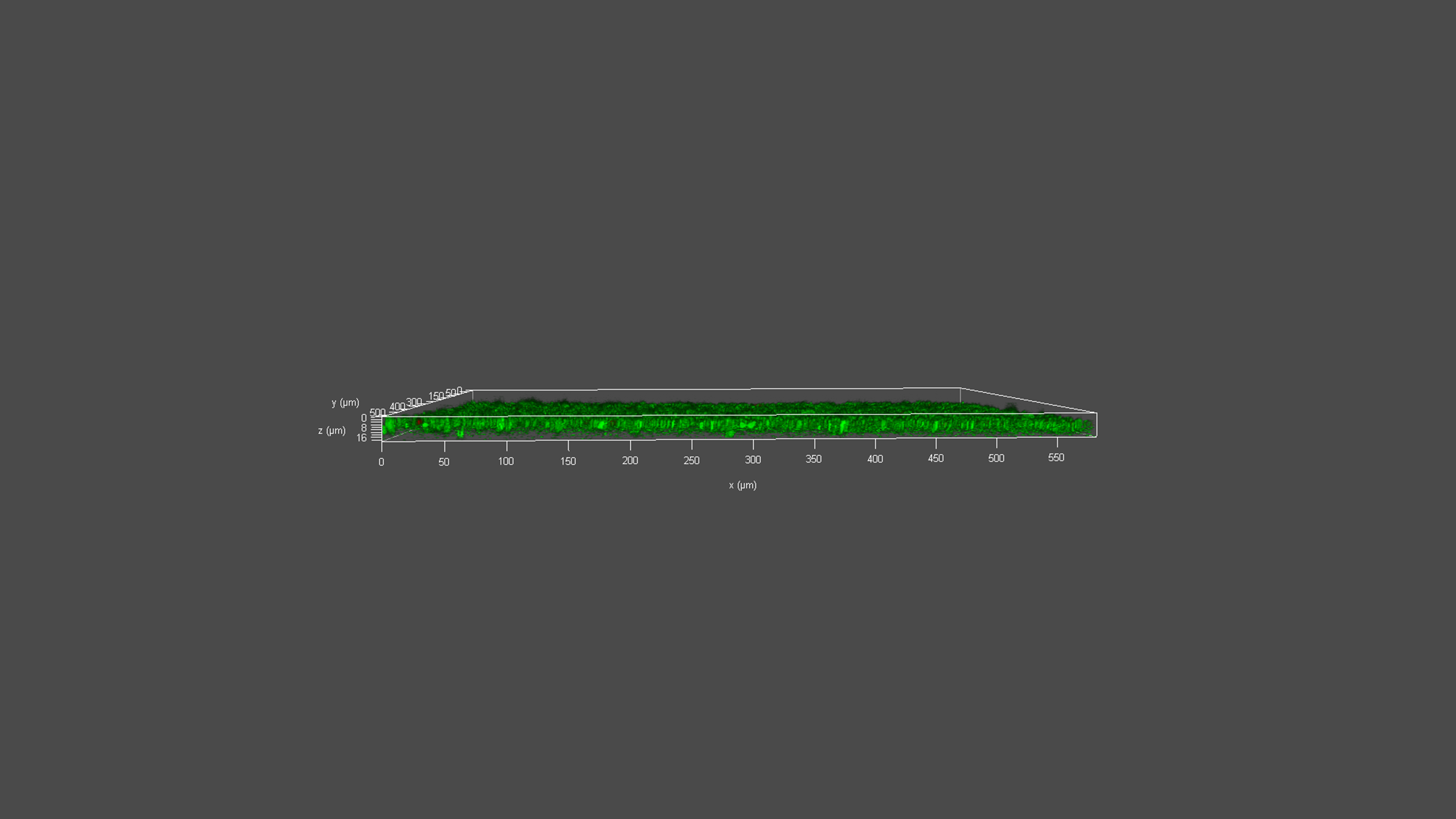

Supplement: Supplementary file 2 — (PNG 898 kb) [file 42770_2021_588_Fig7_ESM.png]

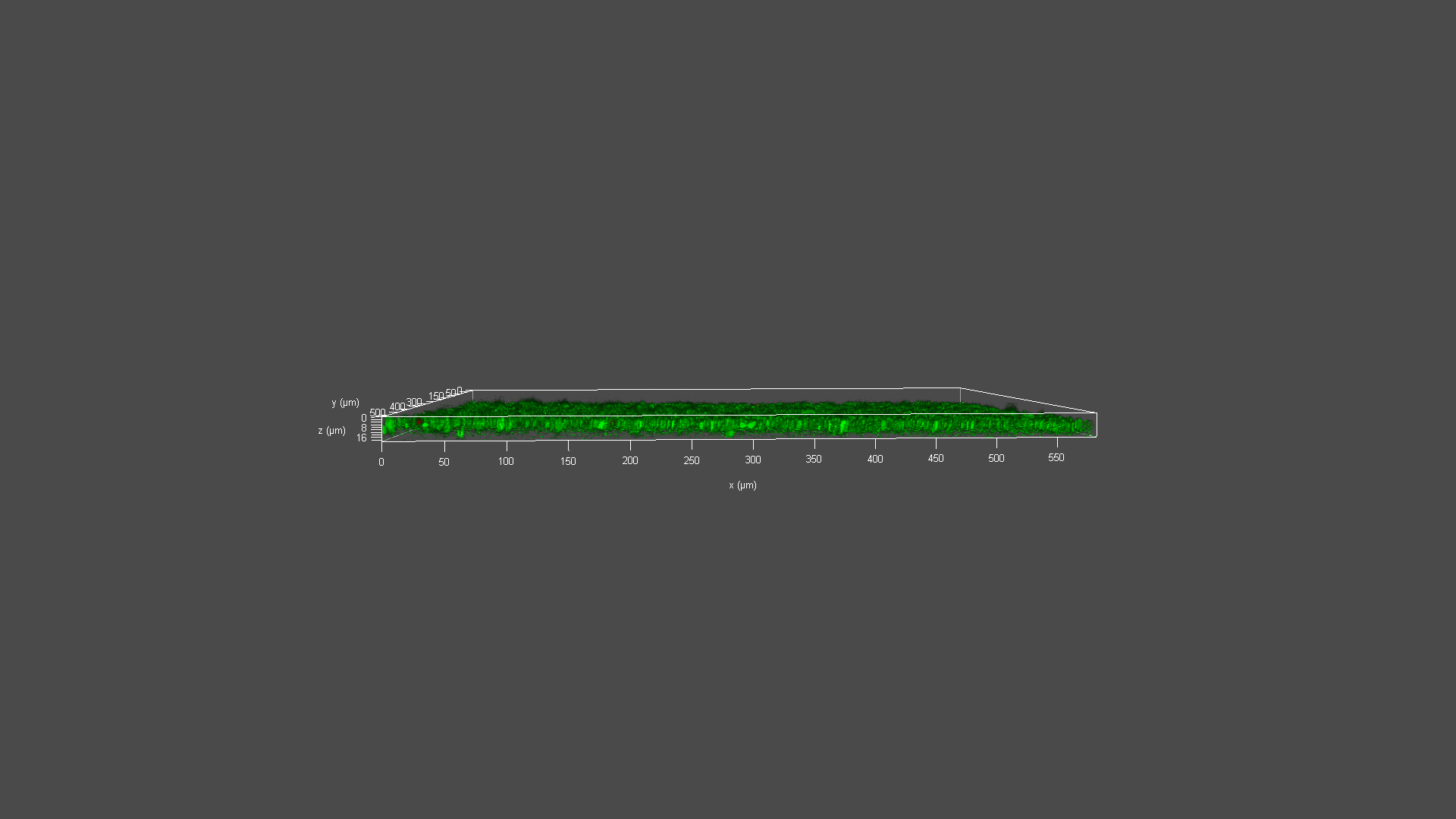

Supplement: Supplementary file 3 — High Resolution Image (TIF 6079 kb) [file 42770_2021_588_MOESM2_ESM.tif]

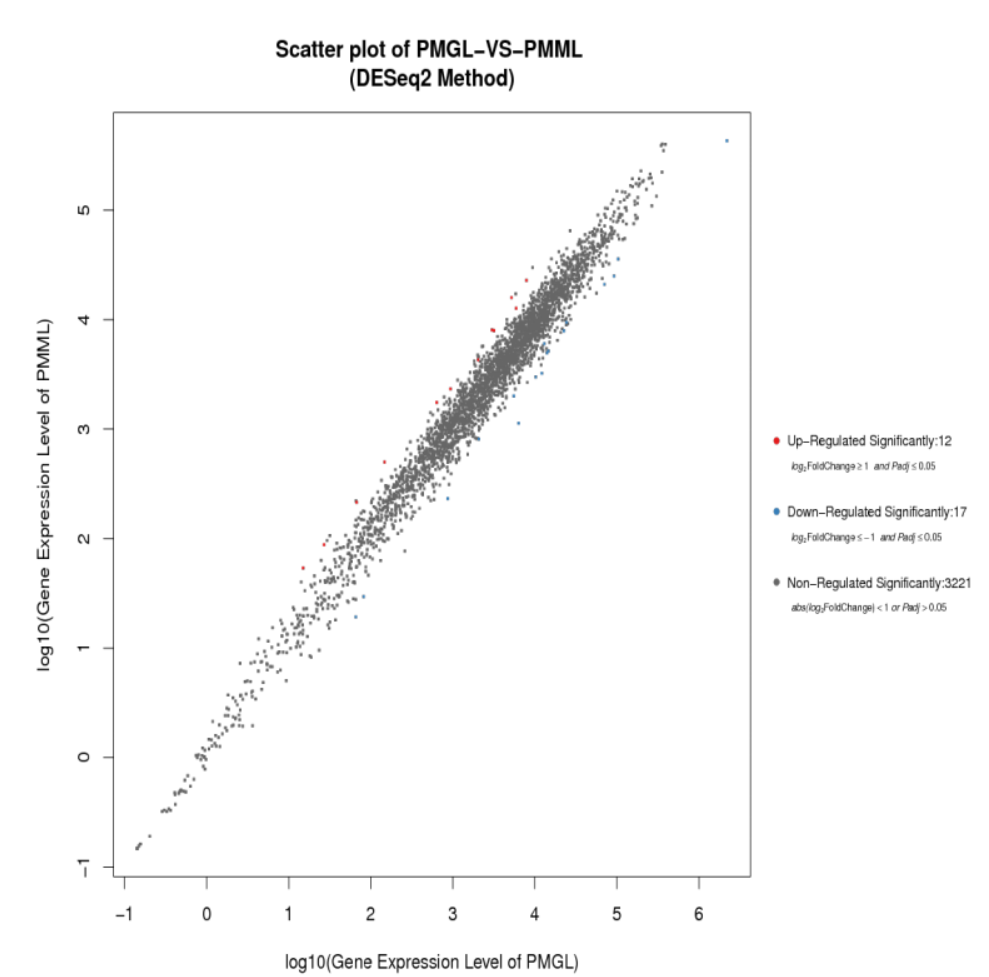

Supplement: Supplementary file 4 — (PNG 121 kb) [file 42770_2021_588_MOESM3_ESM.png]

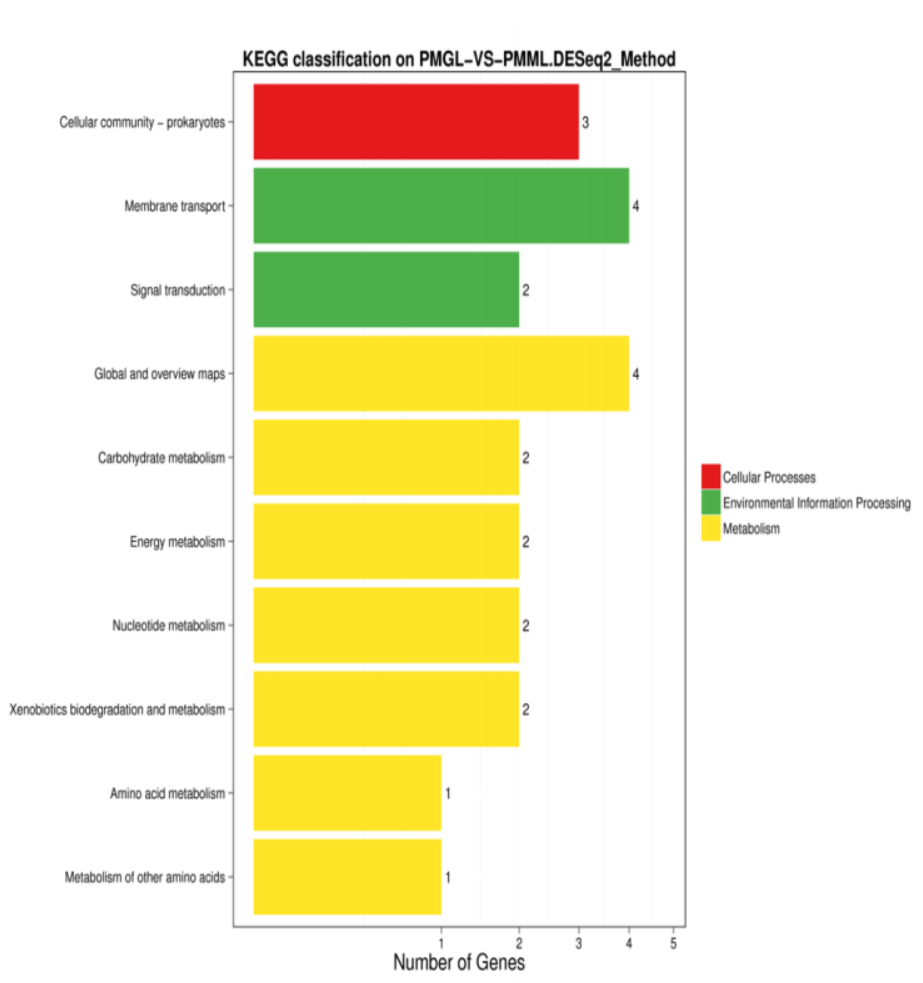

Supplement: Supplementary file 5 — (PNG 108 kb) [file 42770_2021_588_MOESM4_ESM.png]

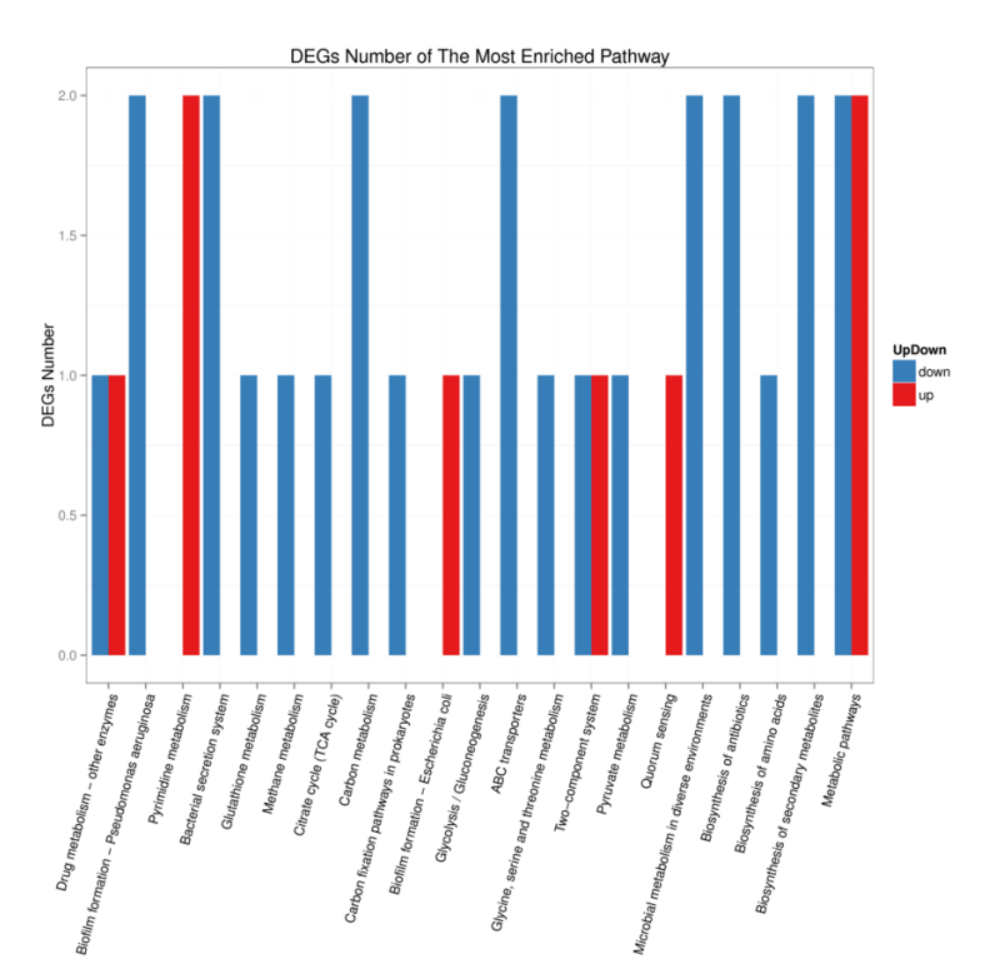

Supplement: Supplementary file 6 — (PNG 154 kb) [file 42770_2021_588_MOESM5_ESM.png]

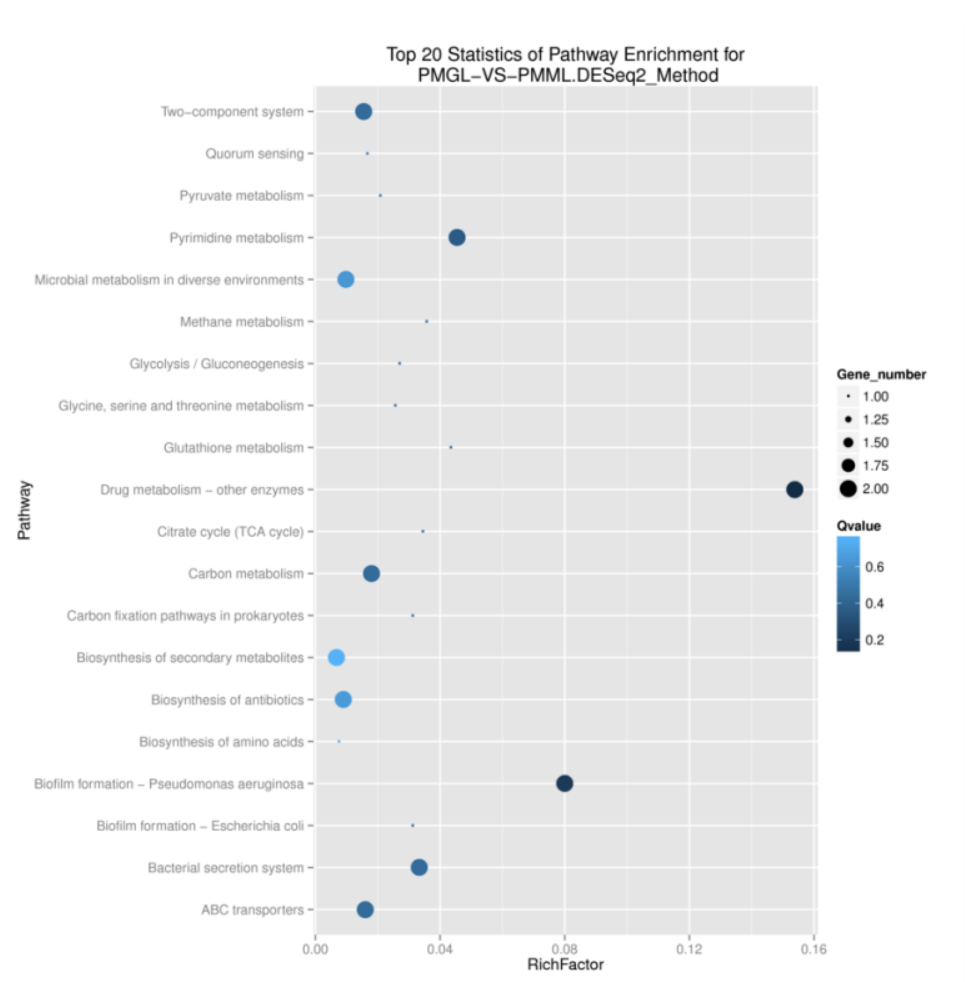

Supplement: Supplementary file 7 — (PNG 143 kb) [file 42770_2021_588_MOESM6_ESM.png]
